# Exploring Ru 4d oxide Ba_2_XRuO_6_ (X = Y, Lu, Sc) for spintronics: pressure-dependent first-principles study

**DOI:** 10.3389/fchem.2026.1814481

**Published:** 2026-09-09

**Authors:** Razia Abdul Majid Qureshi, Altaf Ur Rahman, Muhammad Sajjad, N. A. Noor, Sohail Mumtaz, A. Ibrahim, A. Laref

**Affiliations:** 1 Department of Physics, RIPHAH International University, Lahore, Pakistan; 2 Institute of Physics, Universidade Federal do Rio Grande do Sul, Porto Alegre, Brazil; 3 Nottingham Ningbo China Beacons of Excellence Research and Innovation Institute, University of Nottingham Ningbo China, Ningbo, China; 4 Department of Physics, University of Sargodha, Sargodha, Pakistan; 5 Department of Chemical and Biological Engineering, Gachon University, Seongnam-si, Republic of Korea; 6 Department of Physics, College of Science, King Khalid University, Abha, Saudi Arabia; 7 Department of Physics and Astronomy, College of Science, King Saud University, Riyadh, Saudi Arabia

**Keywords:** elastic properties, ferromagnetic, figure of merit, optical properties, Ru-based double perovskites

## Abstract

Recent advances in the precise control of electromagnetic and transport properties, and the miniaturization of electronic devices, have introduced new prospects in the development of practical quantum computing. Double perovskites have gained particular attention in spintronic applications due to their structural stability, non-toxicity, and inherent spin polarization. In this study, we explore the effect of Ru 4*d*-electrons on the magnetic properties of Ba_2_XRuO_6_ (X = Y, Lu, Sc) using first-principles calculations within the WIEN2k framework. The calculated lattice constants for Ba_2_XRuO_6_ (X = Y, Lu, Sc) using PBEsol-GGA are comparable to reported experimental values. The calculated formation energies (ΔH_f_) of −2.88, −2.61, and −2.43 eV (for X = Y, Lu, and Sc, respectively) confirm thermodynamic stability. Band structure and density of states analyses reveal that all compounds are ferromagnetic semiconductors. All compounds exhibit ferromagnetic semiconducting behavior, and the bandgap increases with pressure up to 4 GPa. The observed magnetism arises from hybridization effects, crystal-field interactions, and exchange coupling. Optical analysis shows strong visible-light absorption, and transport parameters evaluated from 300 to 800 K highlight notable thermoelectric potential. These findings establish Ba_2_XRuO_6_ (X = Y, Lu, Sc) as promising candidates for spintronic and thermoelectric applications.

## 1 Introduction

Nowadays, the exploitation of electron spin in spintronic devices represents a significant advancement toward miniaturized, energy-efficient electronics. Materials exhibiting magnetic response are particularly attractive for spintronics, offering higher energy efficiency compared to conventional semiconductors (Incorvia et al., 2024; Kazim et al., 2024; Tang and Zhu, 2022). Advances in spintronic technology enable the manipulation of electronic spin for data storage and processing, emphasizing the need for materials that optimize energy consumption in device operation. In this context, the discovery and development of double perovskite materials have emerged as highly significant for next-generation energy technologies (Ramawat et al., 2023). These materials possess unique structural features and electronic properties that enable optimal performance in energy storage and conversion devices, including solar cells, fuel cells, and batteries, positioning them as potential frontrunners in the future energy production landscape (Yi et al., 2023).

Double perovskites of the A_2_BB′O_6_ structural family have garnered considerable research interest due to their intriguing magnetic, optical, and thermoelectric properties with ultralow lattice thermal conductivity (Sajjad et al., 2020), which enable diverse technological applications (Zada et al., 2024). Their structural versatility allows tuning of magnetic behavior based on the cation at the A-site, facilitating ferromagnetic, antiferromagnetic, or more complex magnetic ordering. Such tunability makes them suitable for applications in magnetic information storage, magnetoelectric devices, and magnetic-field-responsive sensors (Jin et al., 2022). In terms of optical properties, intercalated double perovskites exhibit favorable electrical conduction and band structures (Leng et al., 2022). Certain compositions with appropriate band gaps demonstrate promise for photovoltaic applications, while their minimal energy losses during light absorption and emission render them ideal for optoelectronic devices and sensors.

Furthermore, double perovskites exhibit excellent thermoelectric performance, with tunable composition and crystal structure (Raihan et al., 2024). They facilitate energy conversion from waste heat, enabling decentralized power generation and contributing to sustainable energy solutions (Amin et al., 2022). Their structural versatility and elemental tunability allow for the design of materials with properties tailored for target applications, including sensors, photovoltaics, fuel cells, and thermoelectric devices.

Previous studies have highlighted the potential of double perovskites for multifunctional applications. Kumar et al. employed density functional theory (DFT) to explore the structural, thermodynamic, magnetic, and optoelectronic properties of Ba_2_YbTaO_6_, revealing features valuable for optoelectronic and magnetic applications (Kumar et al., 2021). Laghzaoui et al. (2022) demonstrated that substitution of technetium with chromium in Sr_2_CrReO_6_ modifies its half-metallic antiferromagnetic behavior, suggesting potential for optoelectronic and thermoelectric applications. Similarly, Shah et al. examined La_2_MTiO_6_ (M = Co, Ni, Cu, Zn) using computational simulations, demonstrating how elemental substitution influences band structure, optical characteristics, and magnetic properties (Shah et al., 2021). Harbi and Moutaabbid applied first-principles calculations to Ba_2_MWO_6_ (M = Mg, Zn, Cd), showing that Ba_2_MgWO_6_ exhibited superior structural, optical, and transport properties, making it a promising thermoelectric candidate (Harbi and Moutaabbid, 2022). Naseri et al. investigated lead-free Ba_2_XSbO_6_ (X = Al, Ga), confirming their direct band gap for photocatalytic applications and favorable thermoelectric performance (Naseri et al., 2023). Theoretical studies of Sr_2_XNbO_6_ (X = La, Lu) further emphasized their high electrical conductivity and thermal transport characteristics, underscoring suitability for optoelectronic and thermoelectric devices (Hanif et al., 2022). Collectively, these studies highlight the versatility and technological potential of double perovskite oxides.

In this study, DFT is employed to investigate the mechanical, magnetic, electronic, optical, and transport properties of ferromagnetic Ba_2_XRuO_6_ (X = Y, Lu, Sc) compounds. We acknowledge that the experimental ground state for these double perovskites is antiferromagnetic with relatively low transition temperatures. However, we wish to clarify that the primary objective of this work is a DFT-based prediction of the electronic and magnetic properties within a specific theoretical framework. Calculated results represent the magnetic ground state as predicted by the exchange-correlation functional (PBEsol-GGA) in this study. Experimental investigations demonstrate antiferromagnetic ground states characterized by distinct Néel temperatures. On the other hand, our DFT simulations indicate a ferromagnetic ground state for the assumed crystal structure. This mismatch may arise from the traditional DFT shortcomings in capturing intricate magnetic exchange interactions and magnetic phenomena at restricted temperatures, the absence of Hubbard’s correction and spin-relativistic effects, or insufficient treatment of electron correlations. Furthermore, the suggested FM ordering should be seen as a theoretical possibility that could be influenced by structural distortions, magnetic frustration, electron correlation effects, or other electronic structure phenomena not entirely addressed by the current computational method. Consequently, the DFT findings suggest a potential pathway to high-temperature ferromagnetism under ideal conditions.

These properties are critical for applications in spintronics and energy conversion devices. Given the absence of prior theoretical or experimental investigations on these compositions, this work aims to uncover novel features and behaviors in double perovskite structures. The findings are expected to enhance the fundamental understanding of these materials, facilitate the design of compounds with tailored properties, and contribute to the development of advanced technological applications.

## 2 Computational methodology

All calculations were performed within the framework of density functional theory (DFT) using the full-potential linearized augmented plane-wave (FP-LAPW) method as implemented in the WIEN2k code (Blaha et al., 2020). Structural optimization and total energy calculations were carried out using the PBEsol generalized gradient approximation (GGA) (Perdew et al., 2008), which is specifically optimized for solid-state systems and provides improved accuracy for lattice constants and bulk properties, particularly in materials containing heavy elements. Since conventional GGA functionals systematically underestimate electronic band gaps, the Tran–Blaha modified Becke–Johnson (TB-mBJ) exchange potential was employed for accurate electronic structure calculations, including band structures and density of states (DOS) (Tran and Blaha, 2009). Spin polarization and spin–orbit coupling (SOC) effects were explicitly included to properly describe the relativistic behavior of Ru 4d electrons and their contribution to the magnetic and electronic properties. Brillouin-zone integrations were performed using a 12 × 12 × 12 Monkhorst–Pack k-point mesh (≈2000 k-points in the full Brillouin zone) for structural, electronic, and magnetic calculations. For transport and thermoelectric property evaluations, a denser 20 × 20 × 20 k-point grid was adopted to ensure numerical accuracy. The space was partitioned into muffin-tin spheres and interstitial regions, with wave functions expanded in spherical harmonics inside the spheres and plane waves in the interstitial region. The plane-wave cutoff was defined by RMT × K_max_ = 8, while the angular momentum expansion inside the muffin-tin spheres was set to ℓmax = 10, and the Fourier expansion cutoff in the interstitial region was fixed at G_max_ = 20. Energy convergence was achieved with a criterion of 10^–3^ mRy. Thermoelectric transport coefficients, including the Seebeck coefficient, electrical conductivity, and electronic thermal conductivity, were computed using the BoltzTraP code within the constant relaxation time approximation (Madsen and Singh, 2006), based on the DFT-derived electronic band structures.

## 3 Results and discussion

### 3.1 Pressure-dependent structural properties

**FIGURE 1:**
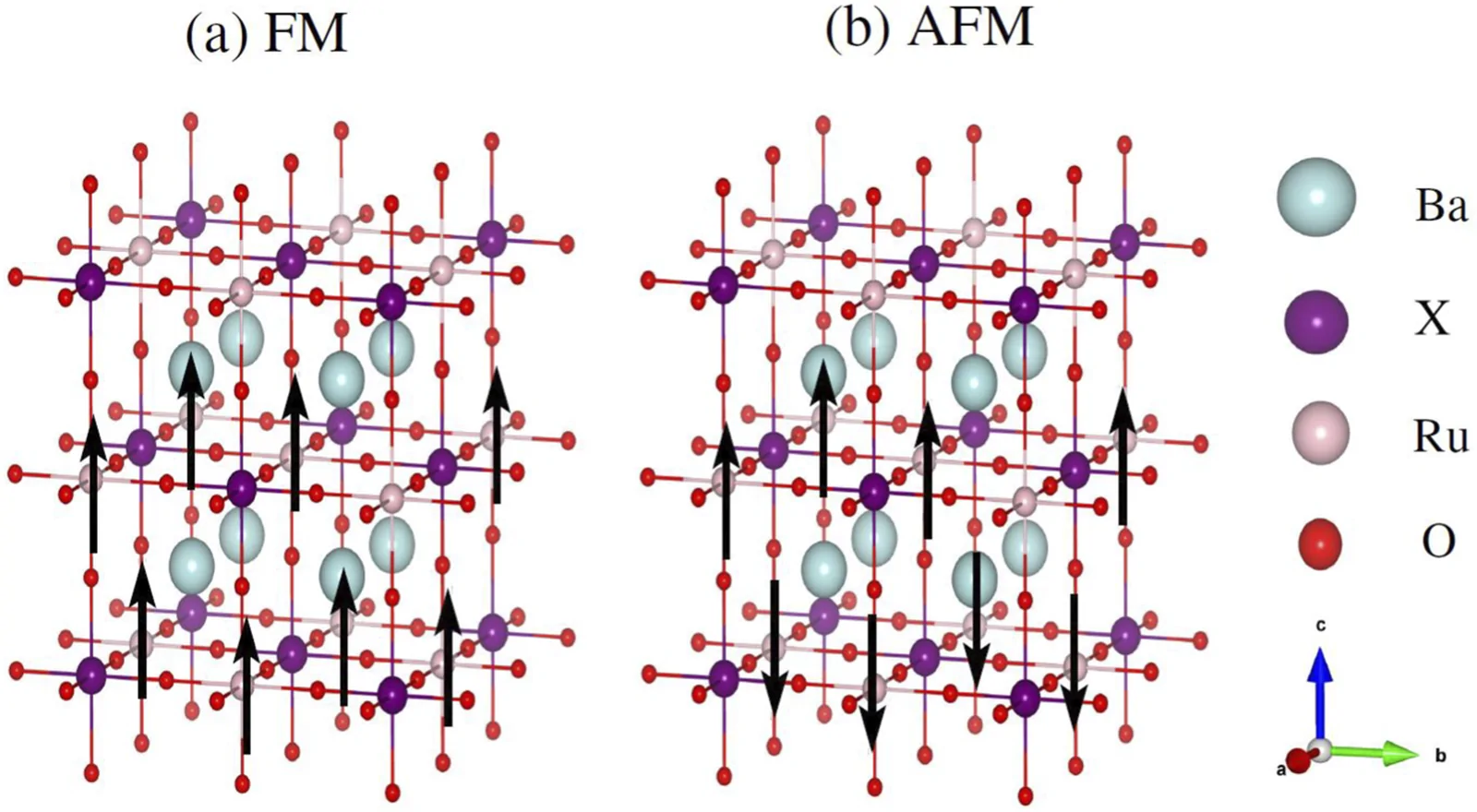
Crystal structure of oxides Ba_2_XRuO_6_ in **(a)** ferromagnetic and **(b)** antiferromagnetic configurations. Light cyan, purple (violet), light pink, and red spheres show Ba, X, Ru, and O atoms, respectively.

The double perovskites Ba_2_XRuO_6_ (X = Y, Lu, Sc) adopt a cubic structure (*Fm* $\bar{3}$ *m* space group) having a number of formula units (Z = 4) in the conventional cubic unit cell as shown in Figure 1. The atomic occupancies in a conventional cubic unit cell are as follows: Ba fills the 8c Wyckoff sites (0.75, 0.25, 0.25); the X cation (Y, Lu, Sc) takes up the 4a Wyckoff position (0, 0, 0); the Ru atom is localized at the 4b Wyckoff site (0.5, 0, 0); while O atoms take up 24e Wyckoff positions (0.75, 0, 0). The energy-volume data are optimized using the Birch–Murnaghan equation, as given below, to obtain lattice parameters (*a*_o_) and bulk modulus (*B*).

$$E\;\left(v\right)=E_{o}\frac{B_{0}}{B^{ˊ}\left(B^{ˊ}-1\right)}\left[V{\left(\frac{V_{o}}{V}\right)}^{B^{ˊ}}\right]+\frac{B_{0}}{B}\left(V-V_{o}\right)$$ (1)

where $E_{o}$ and $B_{0}$ are the equilibrium energy and bulk modulus, respectively, and the derivative of the bulk modulus is given as $B^{ˊ}$ . The calculated equilibrium a_0_ and B_0_ are summarized in Table 1. Using Equation 1, our optimized a_0_ values for Ba_2_YRuO_6_, Ba_2_LuRuO_6_, and Ba_2_ScRuO_6_ (8.31 Å, 8.24 Å, and 8.11 Å, respectively) agree well with reported experimental data (Battle and Jones, 1989; Kayser et al., 2017). The decrease in a_0_ from Y to Sc is attributed to the smaller ionic radii of Sc. The formation enthalpies (ΔH_f_) are all negative, confirming thermodynamic stability. Notably, the formation energy is most negative for Ba_2_YRuO_6_, consistent with (Cai et al., 2017).

**TABLE 1:** Calculated lattice parameters (a_o_) and bond lengths at different pressures, bulk modulus (B_o_), ground-state energy difference (ΔE), and formation energy (ΔH_f_) for Ba_2_XRuO_6_ (X = Y, Lu, Sc).

Compounds	Pressure (GPa)	*a*_*o*_ (Å)	B_o_ (GPa)	ΔE = E_AFM_-E_FM_ (meV)	ΔH_f_ (eV)	Bond length (Å)
Ba_2_YRuO_6_	0	8.31	155.79	42	−2.88	Ba-O 3.14795
						Y-O 2.22646
						Ru-O 2.62549
	2	8.28	​	​	​	Ba-O 3.13659
						Y-O 2.21843
						Ru-O 2.61602
	4	8.25	​	​	​	Ba-O 3.12521
						Y-O 2.21039
						Ru-O 2.60654
Exp.	​	8.33^a^	​	​	​	​
Ba_2_LuRuO_6_	0	8.24	159.4	38	−2.61	Ba-O 3.14795
	​	​	​	​	​	Lu-O 2.57749
	​	​	​	​	​	Ru-O 2.22646
	2	8.21	​	​	​	Ba-O 3.13649
	​	​	​	​	​	Lu-O 2.56810
	​	​	​	​	​	Ru-O 2.21835
	4	8.18	​	​	​	Ba-O 3.12503
​	​	​	​	​	​	Lu-O 2.55871
​	​	​	​	​	​	Ru-O 2.21024
Exp.	​	8.27^a^	​	​	​	​
Ba_2_ScRuO_6_	0	8.11	160.87	35	−2.43	Ba-O 3.14795
	​	​	​	​	​	Sc-O 2.22646
	​	​	​	​	​	Ru-O 2.42029
	2	8.08	​	​	​	Ba-O 3.14795
	​	​	​	​	​	Sc-O 2.22646
	​	​	​	​	​	Ru-O 2.42029
	4	8.05	​	​	​	Ba-O 3.14795
​	​	​	​	​	​	Sc-O 2.22646
​	​	​	​	​	​	Ru-O 2.42029
Exp.	​	8.12^b^	​	​	​	​

^a^ Battle and Jones, 1989.

^b^ Kayser et al., 2017.

**FIGURE 2:**
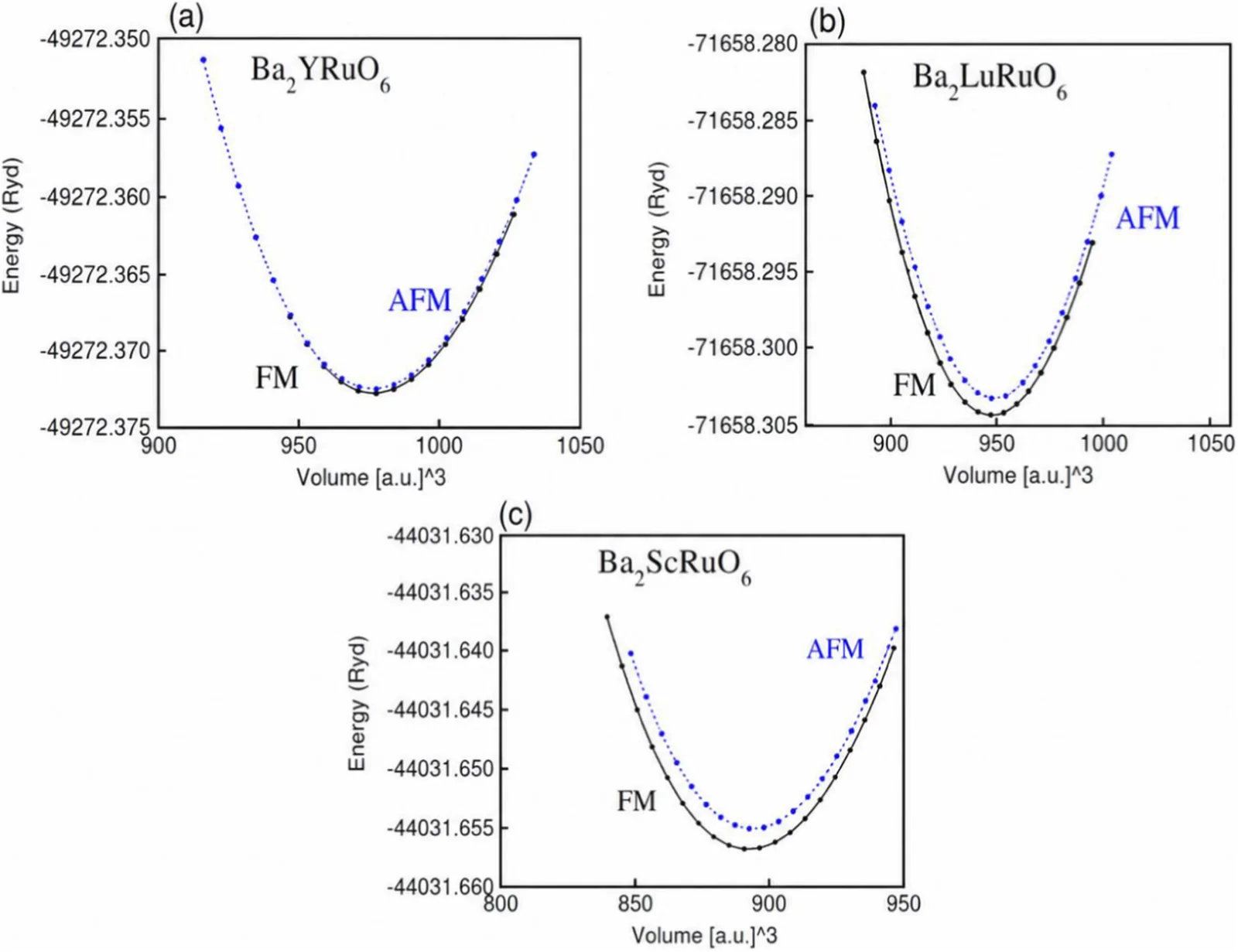
Energy vs. volume plots of **(a)** Ba_2_YRuO_6_, **(b)** Ba_2_LuRuO_6,_ and **(c)** Ba_2_ScRuO_6_ in ferromagnetic (FM) and antiferromagnetic (AFM) phases.

Ferromagnetic ordering above room temperature is a fundamental requirement for materials intended for spintronic applications. The Curie temperature (T_n_) was estimated using the Heisenberg exchange model, expressed as

$$T_{c}=\frac{2\Delta E}{3k_{B}},\text{whether }\Delta E=E_{AFM}-E_{FM},$$

and the corresponding values are reported in Table 1 (Aczel et al., 2022). The calculated Curie temperatures for Ba_2_YRuO_6_, Ba_2_LuRuO_6_, and Ba_2_ScRuO_6_ are 325 K, 294 K, and 271 K, respectively, indicating robust ferromagnetic behavior well above ambient temperature. The total energy as a function of unit-cell volume, obtained within the GGA framework (Figure 2), further demonstrates that the ferromagnetic configuration is energetically more favorable than the antiferromagnetic state for all three compounds (Mahmood et al., 2016). This energetic preference arises from the lower total energy of the ferromagnetic phase, confirming its thermodynamic stability and reinforcing the suitability of these materials for practical spintronic applications (Zhang and Yan, 2010).

The pressure-dependent lattice constants of the studied oxides Ba_2_YRuO_6_, Ba_2_LuRuO_6_, and Ba_2_ScRuO_6_ indicate that the lattice parameter decreases systematically with pressure, whereas the bond lengths obtained via calculation are almost constant. In the case of Ba_2_YRuO_6_, the bond lengths of Ba-O, Y-O, and Ru-O are about 3.15 Å, 2.23 Å, and 2.63 Å, respectively, and exhibit very little change with the applied pressure between 0 and 4 GPa. The same trend of Ba_2_LuRuO_6_, with Ba-O, Lu-O, and Ru-O bonds being constant. The bond lengths of the Ba-O, Sc-O, and Ru-O in Ba_2_ScRuO_6_ do not change under pressure either, with the bond lengths of Ba-O, Sc-O, and Ru-O of the material having a minimum value of 3.15 Å, 2.23 Å, and 2.42 Å. This dissimilarity of bond lengths, even when the lattice is contracted, indicates that the structural framework can adjust itself to the pressure by small alterations in bond angles and tilting of octahedra instead of compressing bonds directly. Also, the fact that the calculated and experimental *a*_*o*_ (Å) are close to each other confirms the accuracy of the structural optimization. Overall, these findings show that these double perovskites have rigid BO_6_ octahedra, which adds to their stability under pressure.

### 3.2 Pressure-dependent mechanical properties

The pressure dependence of the mechanical behavior of the investigated compounds was analyzed through the calculated elastic constants C_11_, C_12_, and C_44_, that shows a clear increase in mechanical rigidity at compressing forces. For the investigated Ba_2_YRuO_6_, the elastic constants get larger with pressure, increasing the bulk modulus (147.37–161.71 GPa), shear modulus, and Young’s modulus, which signifies enhanced resistance to volume and shape deformation (see Table 2). Mechanical stability of cubic crystals was evaluated using the Born stability criteria, which require the conditions (C_11_ – C_12_ > 0, C_44_ > 0, C_11_ + 2C_12_ > 0, and C_12_ < B_o_ < C_11_) to be satisfied (Ji et al., 2015). All studied compounds fulfill these criteria, confirming their mechanical stability. From these elastic constants, the bulk modulus (B_0_), shear modulus (G), and Young’s modulus (Y) were derived and are summarized in Table 2. Among the three systems, Ba_2_LuRuO_6_ exhibits the highest values of B_0_, G, and Y, indicating comparatively greater stiffness and resistance to elastic deformation even at different pressures.

**TABLE 2:** Calculated pressure-dependent elastic constants (C_11_, C_12_, C_44_), bulk modulus (B), Shear modulus (G), Young’s modulus (Y), Poisson’s ratio (υ), and Pugh ratio ((B_0_/G) of Ba_2_XRuO_6_ (X = Y, Lu, Sc).

Compounds	Pressure (GPa)	C_11_ (GPa)	C_12_ (GPa)	C_44_ (GPa)	B_0_ (GPa)	G (GPa)	Y (GPa)	υ	B_0_/G
Ba_2_YRuO_6_	0	296.08	73.01	59.42	147.37	76.67	196.02	0.2783	1.922
	2	309.41	83.85	66.62	159.03	82.37	210.73	0.2784	1.924
	4	332.02	76.55	71.72	161.71	90.55	228.92	0.2641	1.786
Ba_2_LuRuO_6_	0	312.87	70.02	60.61	151.01	80.37	204.78	0.273	1.878
	2	317.60	80.21	78.49	159.34	92.69	232.92	0.260	1.719
	4	327.53	89.29	77.12	168.70	91.85	233.23	0.269	1.836
Ba_2_ScRuO_6_	0	290.55	86.64	79.92	154.61	88.10	222.14	0.261	1.760
	2	287.48	99.36	86.89	162.07	89.69	227.17	0.266	1.806
	4	331.44	89.95	82.49	170.45	96.12	242.74	0.262	1.773

The ductile or brittle nature of the materials was assessed using the Pugh ratio (B_0_/G) and Poisson’s ratio (υ). All compounds show B_0_/G values exceeding 1.75, which classifies them as ductile materials. This conclusion is further supported by their Poisson’s ratios, which are all greater than 0.26, with values of 0.278 (Ba_2_YRuO_6_), 0.273(Ba_2_LuRuO_6_), and 0.261 (Ba_2_ScRuO_6_) at 0 GPa, confirming dominant ductile behavior. Among all oxides, Ba_2_LuRuO_6_ portrays a general tendency of mechanical stiffness increasing with pressure, as indicated by the increased bulk and shear moduli, albeit with slight changes in elastic constants, which indicates slight structural changes. Its B/G ratio is continuously ductile, and the moderate Poisson ratio of the material is helpful in indicating mixed ionic-covalent bonding behavior. The mechanical behavior of Ba_2_YRuO_6_, Ba_2_LuRuO_6_, and Ba_2_ScRuO_6_ under pressure is commonly like that observed earlier in experimental and theoretical studies of elastic response to pressure. For instance, Li et al. reported that C_11_, C_12,_ and the bulk modulus exhibit systematic pressure dependence for cubic LaBeH_8_, while C_44_, the shear modulus, and Young’s modulus show more complicated pressure dependence (Li et al., 2026). Similarly, Kang et al. showed that for ZrB_2_, pressure increases the elastic stiffness and alters ductility and bonding properties of the material (Kang et al., 2024). Therefore, in the present double perovskites, the increase is seen under compression, indicating greater resistance to deformation as well as pressure-induced stiffening for the elements C_11_, B, G, and Y. All these findings of mechanical parameters prove that pressure increases the mechanical strength of all three compounds, and Ba_2_LuRuO_6_ is the most sensitive to compression. Elastic anisotropy was quantified using the anisotropy factor A, defined as:

$$A=\frac{2C_{44}}{C_{11}-C_{12}}$$ (2)

Using Equation 2, the calculated anisotropy factors are 0.88, 1.02, and 1.08 for Ba_2_XRuO_6_ with X = Y, Lu, and Sc, respectively, indicating that all compounds exhibit elastic anisotropy. A value of A = 1 corresponds to ideal elastic isotropy; therefore, the deviation of A from unity confirms the anisotropic mechanical response of these materials. The complete set of elastic and anisotropy parameters is presented in Table 2.

**FIGURE 3:**
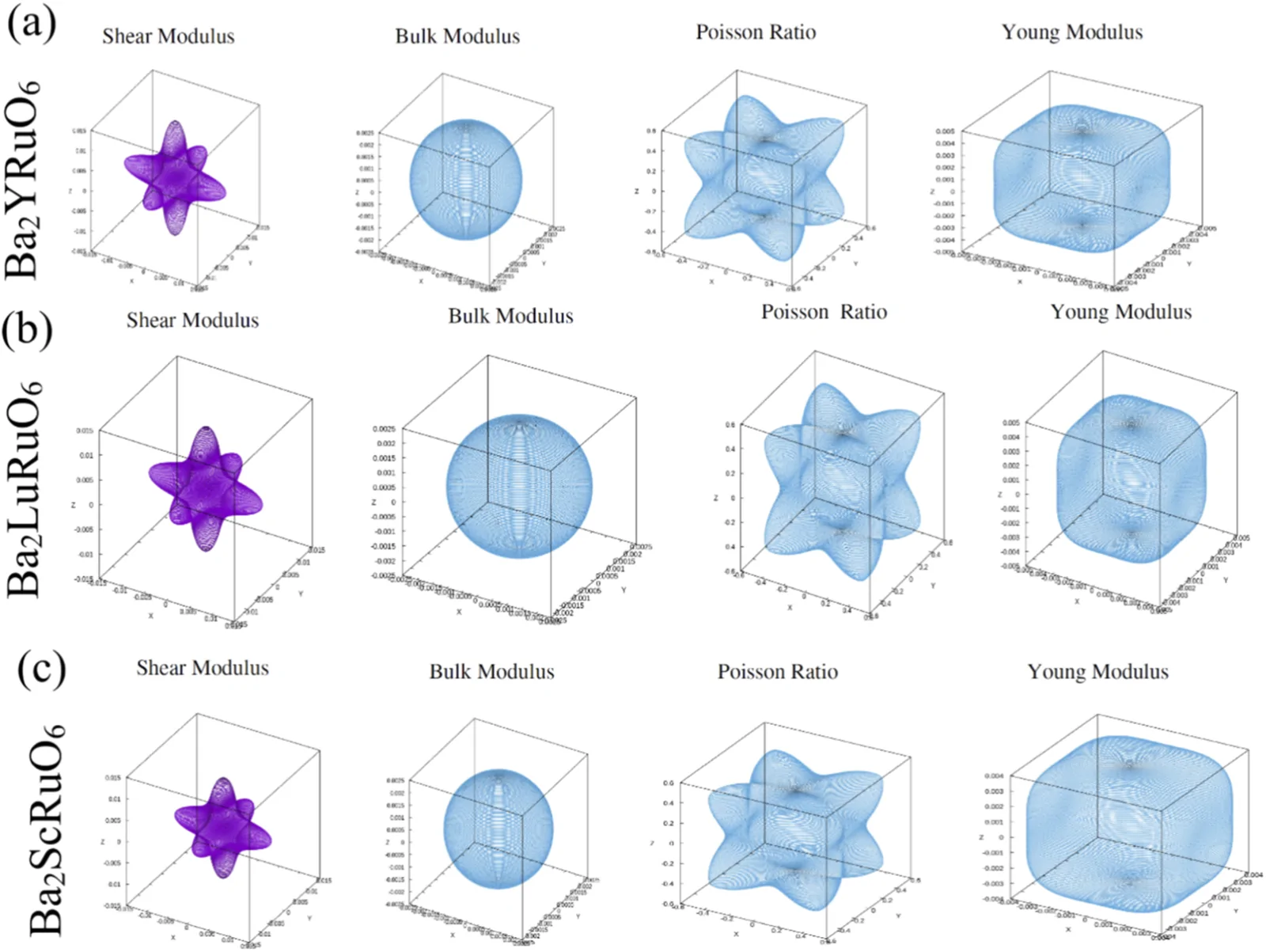
3D representation of Young’s modulus (Y), shear modulus (G), Poisson’s ratio (*v*), and Bulk modulus for **(a)** Ba_2_YRuO_6_, **(b)** Ba_2_LuRuO_6,_ and **(c)** Ba_2_ScRuO_6_.

To analyze elastic anisotropy, three-dimensional directional surface plots of the bulk modulus (B), Young’s modulus (Y), shear modulus (G), and Poisson’s ratio (υ) were generated (Figure 3) at 0 GPa. The bulk modulus exhibits an almost spherical distribution, indicating an approximately isotropic compressional response. In contrast, the distributions of G, Y, and ν deviate significantly from spherical symmetry, revealing pronounced elastic anisotropy in Ba_2_XRuO_6_ (X = Y, Lu, Sc). This anisotropic behavior is further evidenced by the variation between the maximum and minimum directional values of G and Y, demonstrating the strong orientation dependence of the mechanical response. The combined analysis of elastic constants, elastic moduli, and anisotropy parameters confirms that Ba_2_XRuO_6_ (X = Y, Lu, Sc) are mechanically stable, intrinsically ductile materials with non-negligible elastic anisotropy, highlighting their potential suitability for applications in energy-related technologies and optoelectronic devices.

### 3.3 Pressure-dependent electronic properties

**FIGURE 4:**
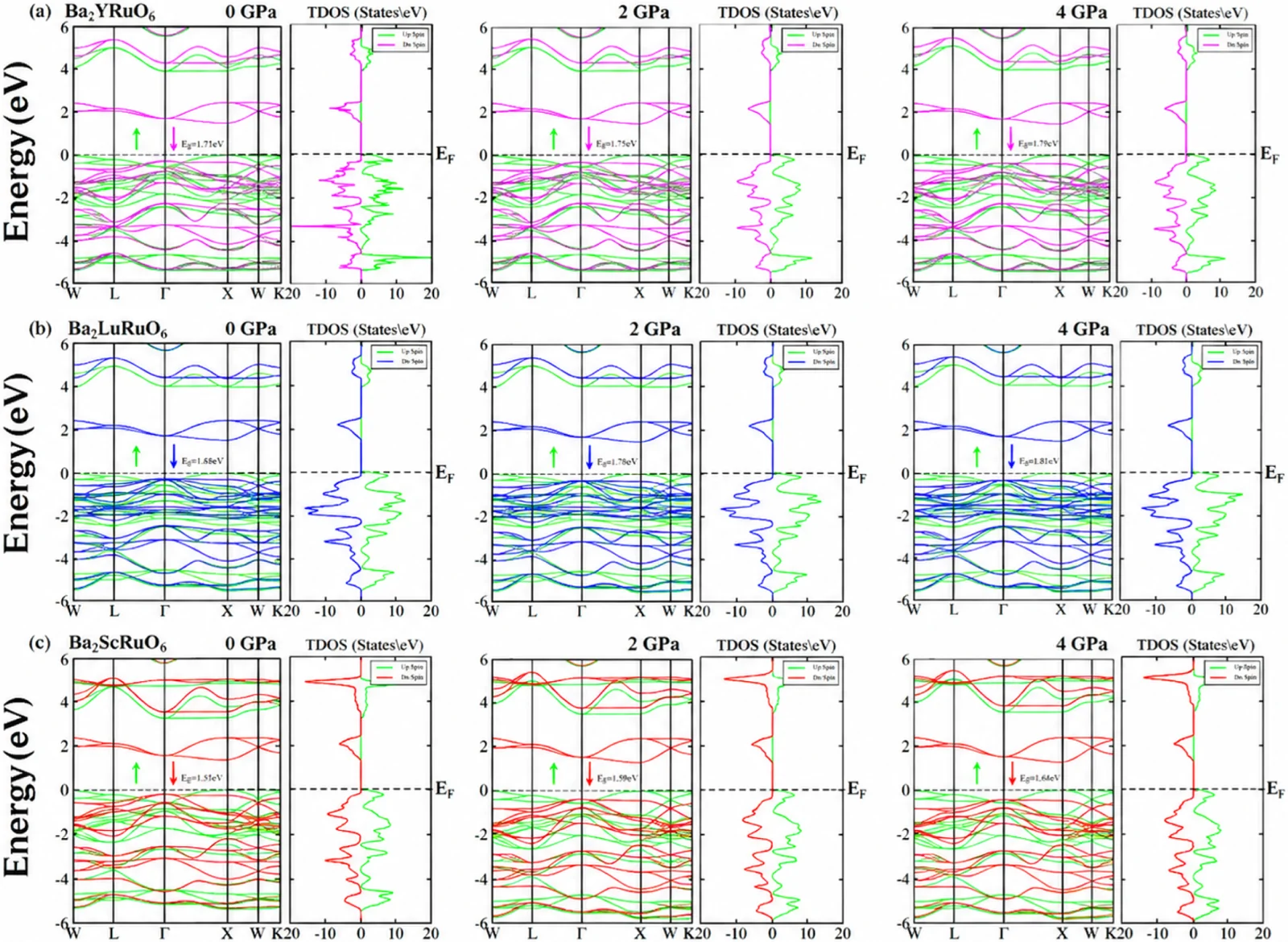
Calculated band structures of **(a)** Ba_2_YRuO_6_, **(b)** Ba_2_LuRuO_6,_ and **(c)** Ba_2_ScRuO_6_ at 0 GPa, 2 GPa, and 4 GPa for spin-up (↑) and spin-down (↓) channels using the mBJ potential.

The investigation of spin-polarized band structures (BS) is essential for understanding the electronic character of Ba_2_XRuO_6_ (X = Y, Lu, Sc). The calculated spin-resolved band structures and corresponding total density of states (TDOS) at pressures up to 4 GPa are presented in Figures 4a–c, providing direct insight into the ferromagnetic electronic behavior of the studied compounds. All three materials exhibit semiconducting behavior with a direct band gap. In the spin-up (↑) channel, the Fermi level (E_F_) lies at the upper edge of the valence band, with no electronic states crossing E_F_, confirming the presence of a band gap. Moreover, the band gap systematically increases with applied pressure up to 4 GPa, indicating pressure-induced band modulation. This clearly establishes a well-defined energy separation between the valence and conduction bands (Soulen et al., 1998).

In contrast, in the spin-down (↓) channel, the Fermi level is located within the energy gap between the valence and conduction bands, further confirming the ferromagnetic semiconducting nature of these compounds. This coexistence of spin polarization and semiconducting behavior highlights their strong potential for spintronic applications, where spin-dependent transport is a key functional requirement (Flemban et al., 2022; Li et al., 2019; Mahmood et al., 2016; Mahmood et al., 2020). Spin-polarized band structure analysis thus provides critical information on both the magnetic ordering and band gap characteristics of these materials.

**FIGURE 5:**
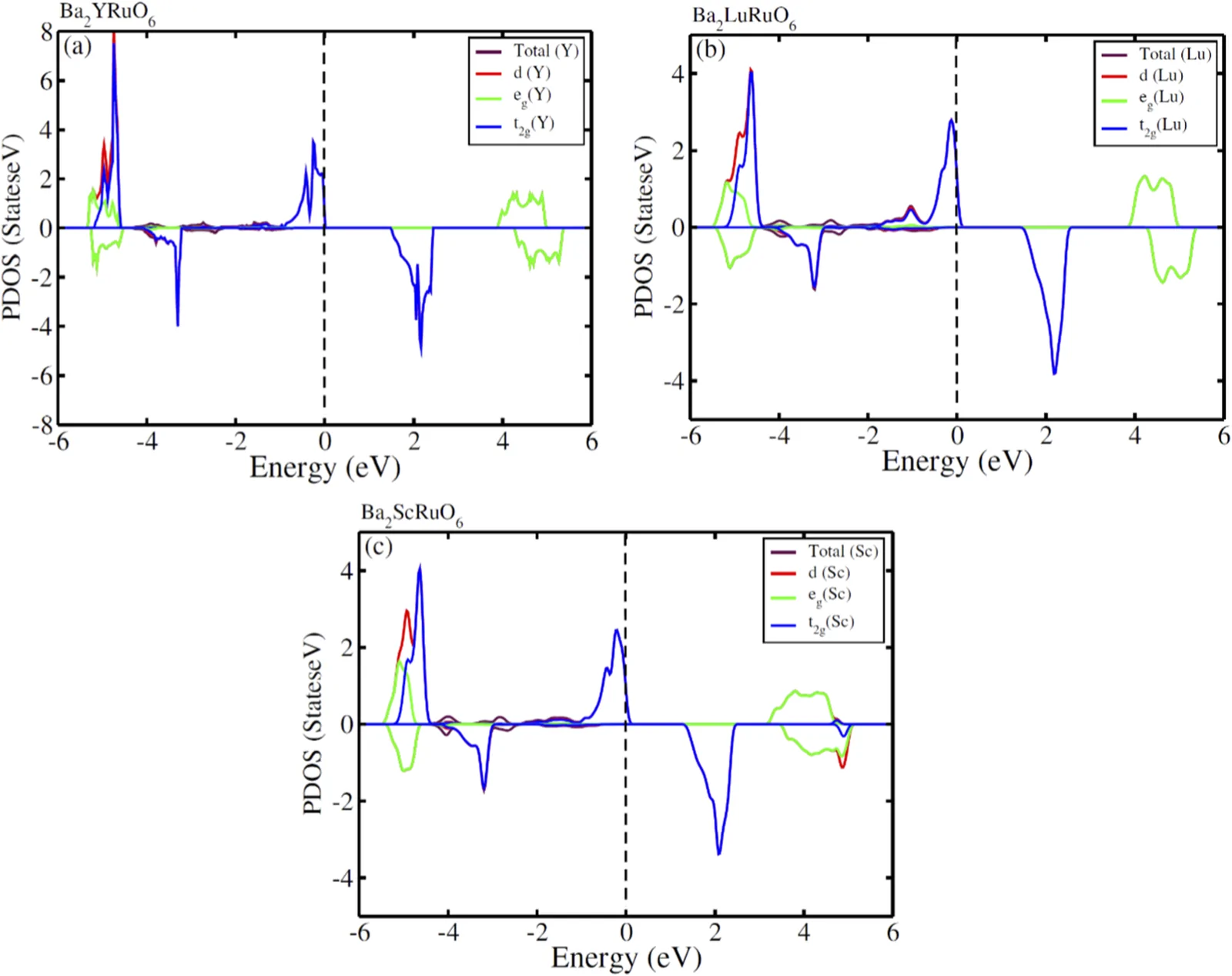
Partial DOS of X-site for **(a)** Ba_2_YRuO_6_, **(b)** Ba_2_LuRuO_6,_ and **(c)** Ba_2_ScRuO_6_ in spin-up (↑) and spin-down (↓) channels at 0 GPa.

For Ba_2_YRuO_6_, Ba_2_LuRuO_6_, and Ba_2_ScRuO_6_, key electronic features, including ferromagnetic ordering, orbital hybridization, exchange interactions, and partial density of states (PDOS), were analyzed and are presented in Figures 5a–c. The density of states (DOS) provides a comprehensive description of the distribution of electronic states over a wide energy range and is fundamental for understanding the electronic structure of these materials. In Ba_2_YRuO_6_, the valence band (VB) edge in the spin-up channel is primarily dominated by Ru 4d–t_2g_ states, with minor contributions from Y 4*d* and O 2*p* orbitals. The central region of the valence band, extending from approximately −1.2 to −3.8 eV, is mainly composed of hybridized Y 4d and O 2*p* states, indicating strong covalent interactions. The conduction band (CB) edge is largely formed by Ru 4*d–e*_*g*_ states, together with contributions from Y 4d orbitals. In contrast, the higher-energy conduction states are mainly associated with Ru 4*d–t*_*2g*_ and Y 4*d* orbitals (Mahmood et al., 2018). Notably, the shifts in peak positions and variations in intensity observed in the DOS spectra for Ba_2_LuRuO_6_ and Ba_2_ScRuO_6_, relative to Ba_2_YRuO_6_, indicate significant modifications in the electronic structure induced by X-site substitution, reflecting changes in orbital hybridization and electronic coupling.

### 3.4 Exchange constant and pressure-dependent magnetic moment

The O 2p orbitals make a significant contribution to the electronic structure of these compounds. In the spin-down (↓) channel, these states are located away from the Fermi level (E_F_), whereas in the spin-up (↑) channel they shift closer to E_F_, indicating their active role in spin-dependent electronic behavior. Notable differences in the density of states (DOS) are observed when comparing Ba_2_ScRuO_6_ with Ba_2_YRuO_6_ and Ba_2_LuRuO_6_, reflecting substantial modifications in orbital hybridization induced by X-site substitution.

In the spin-up configuration, the conduction band (CB) edge is primarily formed by Ru 4*d–eg* states together with contributions from Sc 3*d* orbitals, while the valence band (VB) edge is mainly governed by Ru 4*d–t*_*2g*_ and Sc 2*p* states. Conversely, in the spin-down configuration, the VB edge is dominated by strong hybridization between Ru 4*d–e*_*g*_ and O 2*p* states, which plays a critical role in defining the band gap magnitude. The hybridization of different orbital states at the band edges facilitates spin-dependent exchange interactions and promotes spin polarization.

**FIGURE 6:**
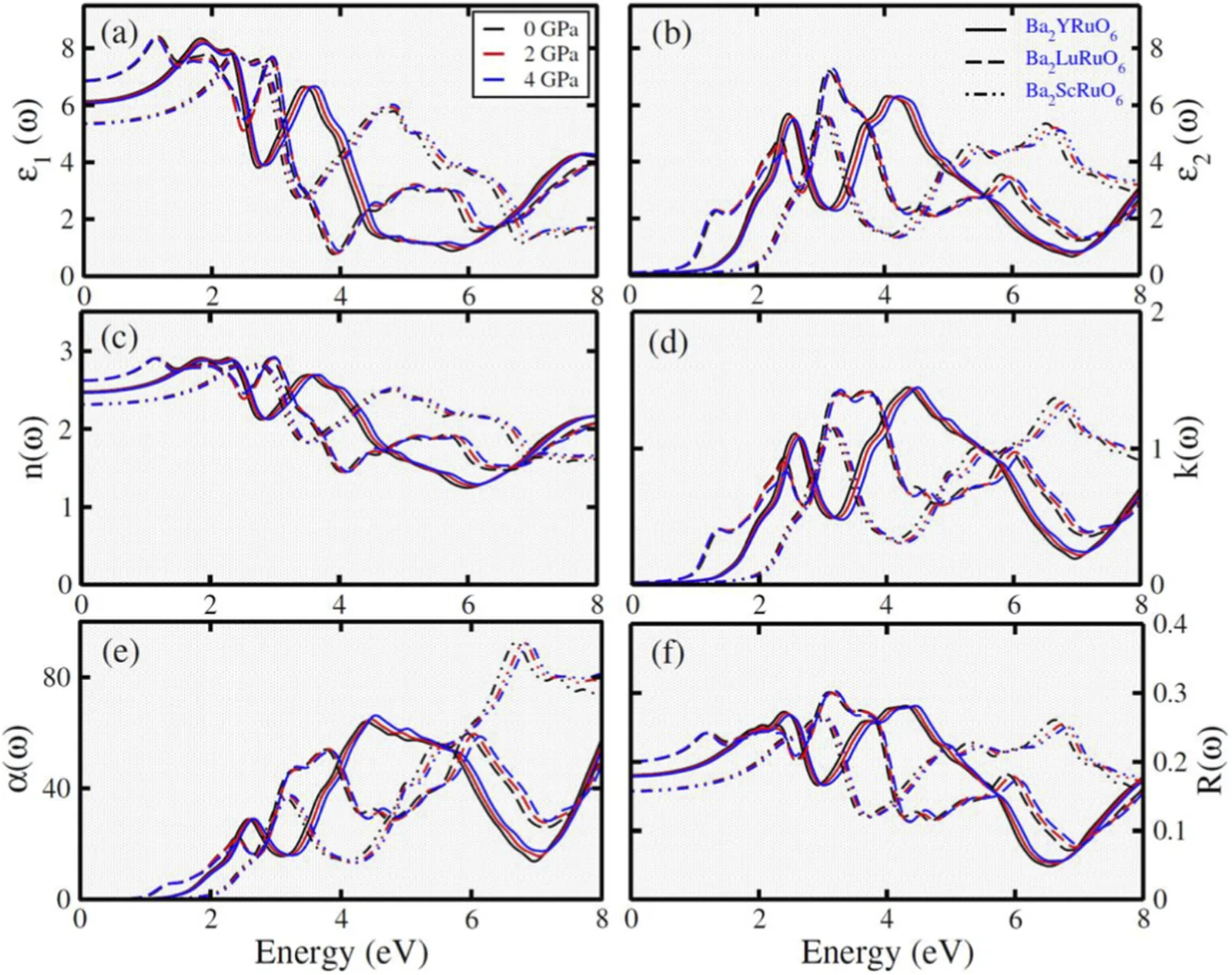
Calculated **(a)** real part ε_1_(ω), **(b)** imaginary part ε_2_(ω), **(c)** refractive index n(ω), **(d)** the extinction coefficient k(ω), **(e)** absorption α(ω), **(f)** reflectivity R(ω) for Ba_2_XRuO_6_ (X = Y, Lu, Sc) at different pressures.

To quantitatively analyze these exchange mechanisms, the crystal-field splitting energy (ΔE_cry_), direct exchange energy (Δ(*d*)), and indirect *p–d* exchange energy (Δ(*pd*)) were evaluated. The indirect exchange term Δ(*pd*) was determined from the energy separation between the *p* and *d* states in the spin-down configuration (Brey et al., 2006) and can also be extracted from the relative positions of the Y/Lu/Sc *p*-states in the spin-down DOS spectra (Figures 4–6). The crystal-field splitting ΔE_cry_ (= E_eg_–E_t2g_) lifts the degeneracy of Ru 4*d* orbitals, driving the *e*_*g*_ (antibonding) states to higher energies and stabilizing the *t*_*2g*_ (bonding) states at lower energies. Ferromagnetic ordering is energetically favored when ΔE_cry_ < Δ(*d*), and the positive value of Δ(d) − ΔE_cry_ indicates that direct exchange dominates the magnetic interaction (see Table 3). The interaction between Ru 4d orbitals and the X-site d orbitals (*4d*/*5d*/*3d* for Y/Lu/Sc, respectively) further contributes to energy exchange and magnetic stabilization. In addition, the negative values of Δ(pd) demonstrate that the spin-down channel enhances ferromagnetic coupling, consistent with indirect exchange-mediated magnetism (Bupu et al., 2017).

**TABLE 3:** Calculated values of field energy (Δ_CF_), Δ_x_(*d*), *p-d* exchange constant (N_o_β), and *s-d* exchange constant (N_o_α) for Ba_2_XRuO_6_.

Compounds	Δ_CF_	Δ_x_(*d*)	Δ_x_ (*pd*)	*N*_*o*_ *α*	*N*_*o*_ *β*
Ba_2_YRuO_6_	4.1	6.7	−0.34	2.58	−0.36
Ba_2_LuRuO_6_	3.8	6.4	−0.28	2.76	−0.29
Ba_2_ScRuO_6_	3.4	6.3	−0.11	1.91	−0.12

It is also observed that the Ru 4*d* states are fully occupied and therefore do not contribute to magnetism. The spin splitting of the conduction band minimum (ΔE_C_) and valence band maximum (ΔE_V_) was quantified using the exchange constants N_0_α and N_0_β, define and calculated by using Equation 3

$$N_{o}\alpha =\frac{{\Delta E\;}_{c}\;}{x\langle S\rangle }\;;\;N_{o}\beta =\frac{{\Delta E\;}_{V}\;}{x\langle S\rangle }$$ (3)

where $\Delta E_{C}=E_{C↓}-E_{C↑}$ and $\Delta E_{V}=E_{V↓}-E_{V↑}$ represent the difference in energy between the down and up spin channels for the minima of the conduction band and maxima of the valence band, respectively. Here, x denotes the magnetic ion concentration, and ⟨S⟩ represents the average magnetic moment per ion. The negative values of N_0_β indicate that the spin-down channel energetically favors ferromagnetism (see Table 3), confirming the dominant role of spin-dependent exchange interactions in stabilizing the magnetic ground state (Kato et al., 2004).

The calculated pressure-dependent magnetic moments for individual atoms, together with the total magnetic moments, are summarized in Table 4. The magnetic behavior of Ba_2_YRuO_6_, Ba_2_LuRuO_6,_ and Ba_2_ScRuO_6_ as a pressure-dependent factor can be explained by the ferromagnetic properties of these materials under external pressure. In the case of Ba_2_YRuO_6_, the total magnetic moment is almost independent of pressure from 0 to 4 GPa (nearly 3.01 μ_B_), thus indicating that the ferromagnetic ordering of the material is very stable against pressure. Although slight changes are observed in the Ru local moment, the ferromagnetic state is maintained, implying that the compression has a weak effect on the exchange interactions. On the same note, Ba_2_LuRuO_6_ also has a nearly fixed total magnetic moment, which is indicative of strong ferromagnetism with small changes in the magnetic moments per atomic site induced by pressure-induced hybridization. By comparison, Ba_2_ScRuO_6_ exhibits a progressive decrease in overall magnetic moment under increasing pressure and is indicative of a reduction in ferromagnetic interactions. This loss is mainly related to a decrease in the Ru magnetic moment, which implies a higher extent of delocalization of the electrons and an enhanced overlap of the electron orbital with the O orbital as compression is applied to the material. Our calculated results indicate that Ba_2_YRuO_6_ and Ba_2_LuRuO_6_ have pressure-insensitive ferromagnetism; Ba_2_ScRuO_6_ has been shown to have a pressure-induced suppression of ferromagnetic ordering, indicating the sensitivity of magnetic exchange interactions to lattice compression and the B-site cation. The magnitude of the magnetic moment is influenced by the Curie temperature (T_c_) and by vacancy distributions at the X and Ru sites. The long-range ferromagnetic ordering in these systems originates from the coupling between localized magnetic moments and itinerant charge carriers, consistent with the Ruderman–Kittel–Kasuya–Yosida (RKKY) interaction mechanism.

**TABLE 4:** Pressure-dependent magnetic moment values for Ba_2_XRuO_6_ (X = Y, Lu, Sc) oxides, total and their local M_Ba_, M_X_, M_Ru_, and M_O_.

Compound	Pressure (GPa)	M_Total_ (μ_B_)	M_Ba_ (μ_B_)	M_X_ (μ_B_)	M_Ru_ (μ_B_)	M_O_ (μ_B_)
Ba_2_YRuO_6_	0	3.00882	0.0014	0.0001	2.8674	0.1470
​	2	3.00882	0.0013	0.0003	2.8535	0.1481
​	4	3.00882	0.0011	0.0005	2.8398	0.1492
Ba_2_LuRuO_6_	0	3.00883	0.0072	0.0008	2.0420	0.1437
​	2	3.00883	0.0076	0.0011	2.0284	0.1448
​	4	3.00801	0.0080	0.0014	2.0147	0.1460
Ba_2_ScRuO_6_	0	2.94410	0.0027	0.0061	1.6928	0.1476
​	2	2.92242	0.0034	0.0066	1.6736	0.1462
​	4	2.89311	0.0039	0.0071	1.6582	0.1449

### 3.5 Pressure-dependent optical properties

The concept of threshold photon energy is fundamental in semiconductor physics, as it governs the onset of optical transitions and plays a central role in determining key optical properties such as absorption, reflection, and transparency in double perovskite oxides under applied pressure. These optical responses are described through the complex frequency-dependent dielectric function, ε(ω), which consists of a real part ε_1_(ω) and an imaginary part ε_2_(ω), expressed as

$$\varepsilon \left(\omega \right)=\varepsilon_{1}\left(\omega \right)+i\varepsilon_{2}\left(\omega \right),$$

where ε_1_(ω) represents the dispersive (polarization) response of the medium, while ε_2_(ω) accounts for optical absorption arising from interband electronic transitions (Penn, 1962). The two components of the dielectric function are intrinsically linked through the Kramers–Kronig relations (given below as Equations 4, 5), which ensure causality and provide a rigorous connection between the absorptive and dispersive optical responses of the material.

$$\varepsilon_{1}\left(\omega \right)=1+\frac{2}{\pi }p\int_{0}^{\theta }\frac{\omega^{\prime }\varepsilon_{2}\left(\omega^{\prime }\right)}{\omega^{\prime 2}-\omega^{2}}d\omega^{\prime }$$ (4)

$$\varepsilon_{2}\left(\omega \right)=-\frac{2\omega }{\pi }p\int_{0}^{\theta }\frac{\varepsilon_{1}\left(\omega^{\prime }\right)}{\omega^{\prime 2}-\omega^{2}}d\omega^{\prime }$$ (5)

here, 𝒫 denotes the Cauchy principal value of the integrals. The dielectric constants describe the intrinsic ability of a material to polarize in response to an external electric field, thereby reflecting its capacity to store and sustain electrical energy. A complete set of optical parameters is essential for understanding the interaction of electromagnetic radiation with matter, including interband transitions, light–matter coupling, and charge-carrier dynamics (Mukherjee et al., 2015).

The optical response of Ba_2_YRuO_6_, Ba_2_LuRuO_6_, and Ba_2_ScRuO_6_ was analyzed through their frequency-dependent dielectric functions and related optical parameters, as illustrated in Figures 6a–f under different pressure conditions. The real part of the dielectric function, ε_1_(ω) (Figure 6a), characterizes the polarization response of the materials under an applied electromagnetic field. The calculated static dielectric constants ε_1_ (0) are approximately 5.0, 5.2, and 5.4 for Ba_2_YRuO_6_, Ba_2_LuRuO_6_, and Ba_2_ScRuO_6_, respectively. These values are consistent with Penn’s model (Penn, 1962),

$$\varepsilon_{1}\left(0\right)\approx 1+{\left(\frac{\hbar \omega_{p}}{E_{g}}\right)}^{2}$$

which predicts an inverse relationship between the dielectric constant and the band gap energy. Accordingly, Ba_2_ScRuO_6_, possessing the smallest band gap, exhibits the largest dielectric constant among the three compounds.

With increasing photon energy, ε_1_(ω) initially decreases, reaching a local minimum in the 3–4 eV range, followed by a secondary enhancement near ∼6 eV, and subsequently a gradual decline toward 8 eV. The positive values of ε_1_(ω) throughout the investigated energy window (0–8 eV) confirm the semiconducting nature of the materials, as no negative ε_1_(ω) values (which would indicate metallic behavior) are observed.

The imaginary part of the dielectric function, ε_2_(ω) (Figure 6b), represents the absorptive response of the system. The ε_2_(ω) spectra display two prominent peaks centered around ∼2.2 eV and 5–6 eV, corresponding to interband electronic transitions from the valence to conduction bands. Below the optical threshold, ε_2_(ω) remains nearly zero, indicating the absence of intraband absorption processes. The peak magnitudes of ε_2_(ω) reach approximately 6.0 for Ba_2_YRuO_6_, 5.5 for Ba_2_LuRuO_6_, and 5.0 for Ba_2_ScRuO_6_, followed by a gradual decrease with increasing photon energy, consistent with the dispersive electronic band structure of these materials (Aziz et al., 2022).

The refractive index n(ω), computed using Equation 3

$$n\left(\omega \right)=\frac{1}{\sqrt{2}}{\left[\sqrt{\varepsilon_{1}^{2}\left(\omega \right)+\varepsilon_{2}^{2}\left(\omega \right)}+\varepsilon_{1}\left(\omega \right)\right]}^{1/2}$$ (6)

provides insight into the propagation of light within the material. As illustrated in Figure 6c, the static refractive index values, obtained from $n\left(0\right)=\sqrt{\varepsilon_{1}\left(0\right)}$ , are approximately 2.2, 2.25, and 2.3 for Ba_2_YRuO_6_, Ba_2_LuRuO_6_, and Ba_2_ScRuO_6_, respectively. Each compound exhibits a pronounced peak in the 2–3 eV range, followed by a gradual decrease at higher photon energies, indicative of normal dispersion behavior. The strong correlation between n(ω) and ε_1_(ω) reflects the dependence of the refractive response on electronic polarization within the crystal lattice.

The extinction coefficient, k(ω), which quantifies the material’s capacity to absorb photons, is determined as Equation 7

$$k\left(\omega \right)=\frac{1}{\sqrt{2}}{\left[\sqrt{\varepsilon_{1}^{2}\left(\omega \right)+\varepsilon_{2}^{2}\left(\omega \right)}-\varepsilon_{1}\left(\omega \right)\right]}^{\frac{1}{2}}$$ (7)

As shown in Figure 6d, k(ω) remains nearly zero below 1.0 eV, indicating optical transparency in the low-energy region. At higher photon energies, k(ω) increases sharply, exhibiting a major peak around 3.0 eV, corresponding to interband transitions observed in ε_2_(ω). The maximum k(ω) values are approximately 1.2, 1.2, and 1.25 for Ba_2_YRuO_6_, Ba_2_LuRuO_6_, and Ba_2_ScRuO_6_, respectively, indicating that Ba_2_ScRuO_6_ demonstrates slightly stronger absorption, consistent with its smaller band gap.

The absorption coefficient, α(ω), which further characterizes photon–electron interactions, is expressed as Equation 8

$$\alpha \left(\omega \right)=\frac{\sqrt{2}\;\omega }{c}{\left[\sqrt{\varepsilon_{1}^{2}\left(\omega \right)+\varepsilon_{2}^{2}\left(\omega \right)}-\varepsilon_{1}\left(\omega \right)\right]}^{\frac{1}{2}}$$ (8)

As depicted in Figure 6e, α(ω) rises sharply around 2 eV and attains maxima against the approximate energy ranges of 2.0, 2.2, and 2.4 eV for Ba_2_ScRuO_6_, Ba_2_LuRuO_6_, and Ba_2_YRuO_6_, respectively. This pronounced absorption in the ultraviolet region arises from intense electronic transitions from valence to conduction bands, predominantly involving Ru–O hybridized orbitals, highlighting the suitability of these materials for ultraviolet detection and related optoelectronic applications.

The reflectivity, R(ω), calculated through Equation 9

$$R\left(\omega \right)=\frac{{\left(n\left(\omega \right)-1\right)}^{2}+k^{2}\left(\omega \right)}{{\left(n\left(\omega \right)+1\right)}^{2}+k^{2}\left(\omega \right)}$$ (9)

is presented in Figure 6f. The spectra display finite static reflectivity values, R (0) ≈ 0.14, 0.15, and 0.16 for Ba_2_YRuO_6_, Ba_2_LuRuO_6_, and Ba_2_ScRuO_6_, respectively, with distinct peaks near 3.0 eV corresponding to the principal _2_(ω) transitions. The overall optical response confirms that all three double perovskites exhibit semiconducting behavior with prominent interband transitions in the visible–UV range. The relatively higher values of ε_1_ (0), n (0), and R (0) indicate enhanced polarizability and stronger light–matter interaction, underscoring the potential of these materials for UV sensing and optoelectronic device applications. Such favorable optical properties can be effectively exploited by integrating the studied compounds with suitable electron- and hole-transport layers that provide optimal band alignment, thereby enabling enhanced charge separation and improved photovoltaic efficiency, as demonstrated in previous studies (Ding et al., 2026; Sajjad et al., 2019).

### 3.6 Thermoelectric properties

Efficient energy conversion using materials necessitates exceptional thermoelectric properties, enabling the direct transformation of heat into electrical energy. Thermoelectric performance and efficiency are typically evaluated using parameters such as the Seebeck coefficient (S), electrical conductivity (σ/τ), power factor (σS^2^/τ), thermal conductivity (κ/τ), and the dimensionless figure of merit (ZT). These quantities are inversely correlated, making their simultaneous optimization challenging, as enhancement of one parameter often leads to the suppression of others, thereby complicating the improvement of the overall figure of merit. Strategies such as incorporating “rattler” atom-containing compounds (Sajjad et al., 2020; Sajjad and Singh, 2021; Sharma et al., 2023) or engineering interfaces through the construction of van der Waals heterostructures (Habiba et al., 2026; Saira et al., 2025; Sajjad and Singh, 2022) have proven effective in reducing lattice thermal conductivity without significantly affecting electronic transport. In recent years, double perovskites have garnered significant attention for their promising thermoelectric performance, particularly due to their inherently low lattice thermal conductivity (Sajjad et al., 2020).

**FIGURE 7:**
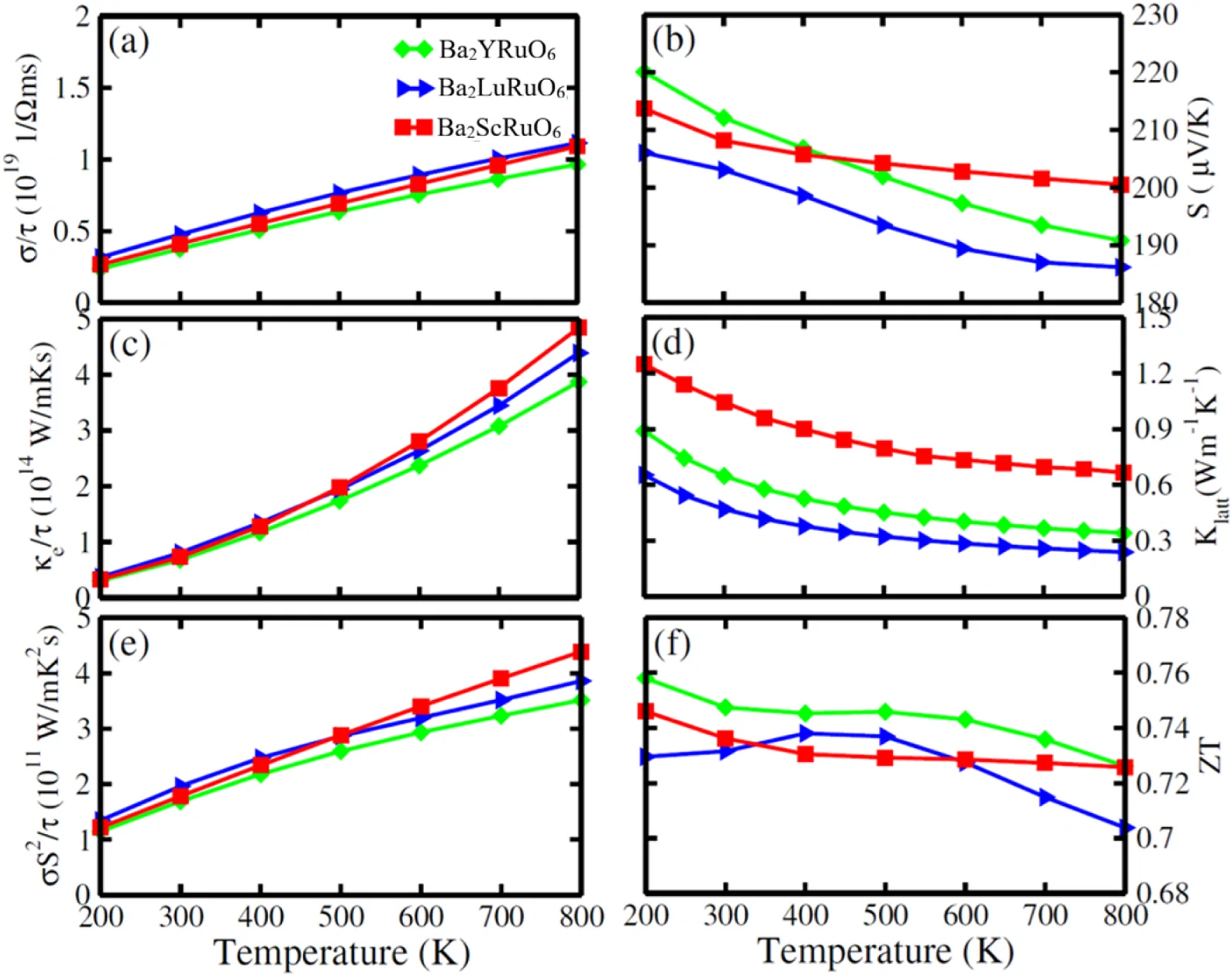
The computed **(a)** electrical conductivity (σ/τ), **(b)** Seebeck coefficients (S), **(c)** electronic thermal conductivity (k_e_/τ), **(d)** lattice thermal conductivity (k_latt_), **(e)** power factor, and **(f)** figure of merit for Ba_2_XRuO_6_ (X = Y, Lu, Sc) as a function of temperature.

In the current study, the thermoelectric properties are calculated using the BoltzTraP code within the WIEN2k framework, with a response time on the order of 10^–14^ s. The electrical conductivity (σ/τ) plays a key role in determining the mobility and concentration of charge carriers, indicating the material type and suitability for specific applications. As illustrated in Figure 7a, σ/τ exhibits an almost linear increase with temperature for Ba_2_YRuO_6_ and Ba_2_LuRuO_6_ in the 200–800 K range, whereas Ba_2_ScRuO_6_ shows a slight linear decrease. Notably, Ba_2_ScRuO_6_ demonstrates relatively higher σ/τ values compared to the other two compounds. At room temperature (∼300 K), σ/τ for Ba_2_ScRuO_6_ is 0.4 × 10^19^ 1/Ω·ms, while Ba_2_YRuO_6_ and Ba_2_LuRuO_6_ exhibit values of 0.3 × 10^19^ 1/Ω·ms and 0.45 × 10^19^ 1/Ω·ms, respectively. The Seebeck coefficient (S) quantifies the voltage response of a material to a temperature gradient. Figure 7b presents the temperature dependence of S over the 200–800 K range. Ba_2_ScRuO_6_, Ba_2_YRuO_6_ and Ba_2_LuRuO show a linear decrease in S with temperature. This behavior can be attributed to variations in charge carrier concentration and mobility; at lower temperatures, the increase in S arises from enhanced carrier mobility due to concentration, while at higher temperatures, thermal effects dominate, restricting mobility or modifying carrier type, resulting in a decreasing S.

The total thermal conductivity (κ/τ) comprises lattice (κ_lat_}) and electronic (κ_e_) contributions. Due to limitations in the BoltzTraP simulations, only κ_e_ is explicitly reported in Figure 7c. As the temperature rises, lattice vibrations intensify, generating additional phonons and enhancing electronic mobility. The electronic thermal conductivity κ_e_ increases consistently with temperature, reaching 0.46/0.5/0.47 × 10^14^ W/m K·s at 300 K and a maximum of 3.9/4.3/4.9 × 10^14^ W/m K·s at 800 K for Sc/Lu/Y, respectively. The ratio κ/σ for all studied compounds is on the order of 10^–4^, indicating their suitability for thermoelectric applications. The lattice thermal conductivity (κ_l_) was independently calculated using the Slack model (Slack, 1973), as shown in Figure 7d. κ_l_ decreases monotonically with temperature, ranging from 1.2 to 0.5 W/m K for Sc/Lu/Y over 200–800 K. Importantly, κ_l_ remains significantly lower than κ_e_, suggesting that lattice contributions do not critically limit overall thermoelectric performance.

The power factor (PF = σS^2^) is a crucial parameter that determines the efficiency of a material in converting a temperature gradient into electrical energy. As depicted in Figure 7e, PF increases with temperature for all Ba_2_XRuO_6_ (X = Y, Lu, Sc), rising from 1.7/1.5/2.0 × 10^11^ W/m K^2^·s at 300 K to 5.0/3.9/4.3 × 10^11^ W/m K^2^·s at 800 K for Sc/Y/Lu, respectively. This highlights the potential of these compounds for high-temperature thermoelectric devices. The dimensionless figure of merit, ZT = σS^2^T/κ, is shown in Figure 7f. From 300 K onward, Ba_2_ScRuO_6_ exhibits a gradual decrease in ZT. This behavior results from the slight reduction in σ/τ relative to S and κ_e_/τ. Such characteristics are desirable for thermoelectric materials intended for high-temperature applications, waste heat recovery, and power generation.

## 4 Conclusion

In this work, we have systematically investigated the structural, mechanical, electronic, magnetic, optical, and thermoelectric properties of the double perovskite oxides Ba_2_XRuO_6_ (X = Y, Lu, Sc). The predicted lattice constants for all compounds are in good agreement with experimental data, confirming the reliability of the structural models. Both antiferromagnetic and ferromagnetic configurations were examined, with results indicating that the ferromagnetic phase is thermodynamically more stable. The calculated formation energies of −2.88, −2.61, and −2.43 eV for Ba_2_YRuO_6_, Ba_2_LuRuO_6_, and Ba_2_ScRuO_6_, respectively, further corroborate the structural stability of these materials. Spin-polarized density of states (DOS) analyses revealed a ferromagnetic semiconducting nature, with Ru–O orbital hybridization contributing significantly to the magnetic moment and high Curie temperature (T_c_). Valence electron hybridization and exchange energy analyses indicate that ferromagnetism in these systems arises primarily from electronic spin polarization rather than clustering effects. Quantum confinement effects were reflected in the negative exchange constants and Δ(*pd*) energy values. Optical property calculations, including the refractive index *n*(*ω*), extinction coefficient *k*(*ω*), absorption coefficient *α*(*ω*), and reflectivity *R*(*ω*), demonstrate that these materials are well-suited for ultraviolet (UV) optoelectronic applications. Thermoelectric analysis shows that the Seebeck coefficient (S) confirms p-type semiconducting behavior, while the power factor (PF) and figure of merit (ZT) increase with temperature, indicating improved thermoelectric performance at elevated temperatures. Overall, the comprehensive results establish that Ba_2_XRuO_6_ double perovskites exhibit robust ferromagnetic semiconducting properties and possess considerable potential for multifunctional applications in spintronic, optoelectronic, and thermoelectric devices.

## Data Availability

The raw data supporting the conclusions of this article will be made available by the authors, without undue reservation.
